# Insights of acupuncture in modulating gut microbiota in stroke treatment

**DOI:** 10.3389/fneur.2025.1579585

**Published:** 2025-06-30

**Authors:** Hang Xing, Wan-juan Lin, Wen-long Hu, Juan-shu Cao, Li-yan Cui, Wen Lyu, Hong-kun Zhu, Ai-jun Wu, Qiu-xia Xu, Yan Zhao, Sheng-yong Bao

**Affiliations:** Department of Rehabilitation, Shenzhen People's Hospital (The Second Clinical Medical College, Jinan University, The First Affiliated Hospital, Southern University of Science and Technology), Shenzhen, Guangdong, China

**Keywords:** stroke, acupuncture, gut microbiota, gut-brain axis, insight

## Abstract

Stroke, an acute neurological disorder caused by the interruption of cerebral blood flow, is the second leading cause of death and a major contributor to long-term disability worldwide. Recent research has increasingly highlighted the role of gut microbiota in stroke recovery. Post-stroke gut microbiota dysbiosis, characterized by an imbalance in microbial composition, exacerbates neuroinflammation and neuronal injury. Restoring the balance of the gut microbiome may facilitate neurological recovery. Acupuncture, a traditional therapeutic modality, has shown potential in stroke rehabilitation. Evidence suggests that acupuncture modulates gut microbiota composition, restores gut barrier integrity, and reduces endotoxin translocation into the bloodstream, which collectively reduces systemic inflammation and alleviates neurological damage. Additionally, acupuncture may influence immune responses through the gut-brain axis, a bidirectional communication system between the gut and brain, thereby suppressing neuroinflammation and promoting neuronal repair. Despite these promising findings, current evidence is limited by methodological inconsistencies, including variability in acupuncture protocols and heterogeneous patient populations, which may confound result interpretation. The precise mechanisms underlying acupuncture's modulation of gut microbiota and its role in stroke recovery remain unclear. Future studies should adopt standardized protocols and larger sample sizes to improve reproducibility, validate these findings, explore the molecular pathways involved, and determine the clinical efficacy of acupuncture in stroke rehabilitation.

## 1 Introduction

Stroke is an acute neurological condition caused by disrupted blood flow to the brain, leading to brain injury. It is typically classified into two main types: ischemic stroke (e.g., cerebral infarction) and hemorrhagic stroke (e.g., intracerebral hemorrhage and subarachnoid hemorrhage) (1–3). Ischemic strokes account for around 80% of cases, while hemorrhagic strokes make up about 20% (4). Stroke is the second leading cause of death worldwide and a major cause of long-term disability.

According to the World Health Organization and epidemiological studies, stroke incidence increases with age, and men are more likely to experience a stroke than women (5). An estimated 15 million people worldwide suffer from a stroke annually, with about one-fifth dying and one-quarter of survivors facing long-term disabilities. This high incidence and disability burden pose significant public health and socioeconomic challenges (1–3).

Stroke symptoms depends on its type and the affected brain region. Common ischemic stroke symptoms include sudden-onset unilateral weakness, numbness, language impairment, visual disturbances, and balance problems (6). Hemorrhagic strokes are often associated with severe headaches, vomiting, and consciousness disturbances. Diagnosis relies on clinical symptoms and confirmed through neuroimaging techniques such as Computed Tomography scans and Magnetic Resonance Imaging, which can quickly differentiate between ischemic and hemorrhagic strokes and assess the severity and location of brain injury (6).

Diagnosis mainly depends on clinical manifestations and imaging results. During the acute phase, the Face, Arm, Speech, Time (FAST) acronym is vital for recognizing stroke symptoms and determining the intervention time window (7).

Stroke treatment includes acute-phase interventions and rehabilitation. In the acute phase, ischemic stroke is typically treated with thrombolysis or mechanical thrombectomy to restore blood flow, while hemorrhagic stroke often requires surgery and medication to control bleeding (8–10). However, despite advances in treatment options, several challenges remain.

Neurofunctional recovery after stroke is one of the main issues in stroke management. Stroke-induced brain injury leads to neuronal death and functional loss, making full recovery challenging (11). Although new pharmacological agents (such as neuroprotective drugs) have shown promise in animal models, clinical outcomes remain suboptimal (12).

Immune regulation is another challenge in stroke treatment. Stroke-induced brain inflammation plays a dual role: initial inflammation may help clear damaged cells, but prolonged inflammation worsens brain injury, leading to irreversible neurological deficits (13, 14). Regulating the immune response and reducing chronic inflammation are crucial areas of ongoing stroke rehabilitation research.

## 2 Literature retrieval and selection

To ensure the thoroughness and scientific validity of this mini review, we performed a systematic literature search in PubMed, Web of Science, and the China National Knowledge Infrastructure databases from inception to the April 30, 2025 using the keywords “acupuncture,” “stroke,” “gut microbiota,” and “gut–brain axis.” Two authors independently screened the studies according to predefined inclusion criteria: (1) clinical or animal studies investigating the effects of acupuncture on gut microbiota in stroke; (2) publication in peer-reviewed journals; and (3) clear relevance to mechanistic insights or therapeutic efficacy. Studies were excluded if they were case reports or lacked mechanistic data. A total of 1,109 records were initially retrieved. After screening, 1,100 records were excluded due to being irrelevant, lacking mechanistic data, or not involving clinical or animal stroke models. Finally, nine studies met the inclusion criteria and were included in the review.

## 3 Gut microbiota: basic principles and physiological functions

Gut microbiota refers to the diverse community of microorganisms residing in the human gastrointestinal tract, including bacteria, viruses, fungi, and archaea (15). These microorganisms play essential roles in health, contributing to immune regulation, metabolic processes, and inflammation (15). First, gut microbiota influences the development and maintenance of the immune system (16, 17). Through interactions between gut microbiota, intestinal epithelial cells, and immune cells, it can modulate both local and systemic immune responses (17). For example, certain beneficial bacteria can regulate T-cell subsets in the intestine, promoting anti-inflammatory responses and maintaining immune homeostasis.

Additionally, gut microbiota plays a crucial role in metabolic processes. It participates in the fermentation of carbohydrates, fatty acids, and proteins, influencing nutrient absorption, energy metabolism, and fat storage (18). Gut microbiota also produces short-chain fatty acids (such as acetate and butyrate), which provide energy to intestinal epithelial cells and have anti-inflammatory effects, helping maintain the intestinal barrier (19).

In recent years, the interaction between gut microbiota and the nervous system has garnered significant attention, leading to the concept of the “gut-brain axis” (20). The gut-brain axis theory proposes that gut microbiota communicates with the brain through neural, immune, and endocrine pathways, influencing brain function, behaviors, emotions, and cognition (21).

The gut-brain axis plays a pivotal role in neurological diseases. Research indicates that dysbiosis (imbalance in the gut microbiota) is closely linked to the onset of neurological diseases, including Parkinson's disease, Alzheimer's disease, and stroke (22). Particularly in the post-stroke phase, changes in the gut microbiota are often associated with incomplete neurological recovery (22). After stroke, gut microbiota undergoes significant alterations, which may exacerbate inflammatory responses and hinder brain tissue repair (23). Specifically, gut microbiota interacts with the immune system, modulating central nervous system inflammation and influencing recovery after stroke.

Studies suggest that post-stroke gut microbiota dysbiosis is directly correlated with impaired neurological recovery (24–26). Restoring gut microbiota balance may help improve neurological recovery, reduce inflammation, and enhance the patient's quality of life (24). Moreover, the gut-brain axis may influence brain neurotransmitter synthesis and transmission, potentially regulating mood and cognitive function in stroke patients. Therefore, modulating the gut microbiota presents a novel therapeutic strategy for stroke rehabilitation, particularly in immune regulation and neuroprotection (25, 26).

In summary, gut microbiota plays a crucial role in the recovery of neurological function after stroke, and acupuncture's potential to regulate gut microbiota provides a new avenue for therapeutic exploration. Although these findings suggest a link between gut microbiota modulation and neurological recovery, causality remains unproven. Current evidence primarily relies on correlational data from animal models or small human trials, lacking mechanistic detail. Future research should use longitudinal designs and multi-omics approaches to confirm causality and identify key microbial taxa or metabolites responsible for therapeutic effects.

## 4 The role of gut microbiota in stroke treatment

Stroke, as an acute neurological condition, induces a series of physiological changes, one of which is the disruption of gut microbiota (22). Studies have shown that after a stroke, patients experience significant alterations in the composition of their gut microbiota (22, 27). Beneficial bacteria, such as *Bifidobacterium* and *Lactobacillus*, decrease, while harmful bacteria, including *Enterobacteriaceae* and *Streptococcus*, proliferate. This dysbiosis weakens the intestinal barrier, allowing endotoxins and harmful substances to enter the bloodstream (22). This process affects both gut health and the central nervous system, exacerbating neuronal damage and functional impairments through systemic inflammation.

The gut microbiota plays a crucial role in regulating immune system functions. When the microbiota becomes dysbiotic, it leads to impaired immune regulation, manifested by reduced immune tolerance and excessive inflammation (22). Research has demonstrated that dysbiosis of the gut microbiota significantly influences the immune response after stroke, particularly promoting neuroinflammation (23, 28). Dysbiosis increases intestinal permeability, allowing immune cells and inflammatory mediators to enter the brain, worsening neurodegenerative changes and injury (23, 28).

Recent studies suggest that gut microbiota may play a pivotal role in stroke recovery (23, 29, 30). By modulating immune responses, especially in the gut-associated lymphoid tissue, gut microbiota could improve neurological function post-stroke (23). The microbiota exerts immune-suppressive and anti-inflammatory effects that reduce persistent inflammation in the nervous system, promoting repair. Moreover, gut microbiota produces metabolites such as short-chain fatty acids, which activate anti-inflammatory pathways and mitigate neural damage after stroke (29, 30).

Both clinical and experimental studies indicate a strong correlation between gut microbiota composition and neurological recovery after stroke (22). Animal models have shown that modifying the gut microbiota can improve cognitive and motor functions post-stroke (22, 31). This mechanism likely works by improving immune responses in the brain, inhibiting excessive inflammation, and promoting neuronal repair (32–34). Clinical studies show that probiotics and microbiota-regulating agents can enhance recovery by improving gut barrier function and reducing systemic inflammation (34). These findings suggest that manipulating the gut microbiota may represent a promising therapeutic approach for enhancing stroke recovery.

In summary, the gut microbiota plays a critical role in immune regulation, inflammation control, and neurological recovery following stroke. Future research should explore how modulating gut microbiota with interventions like probiotics or fecal microbiota transplantation can accelerate stroke rehabilitation and offer new therapeutic approaches.

## 5 Research progress on acupuncture in modulating gut microbiota

**Figure 1:**
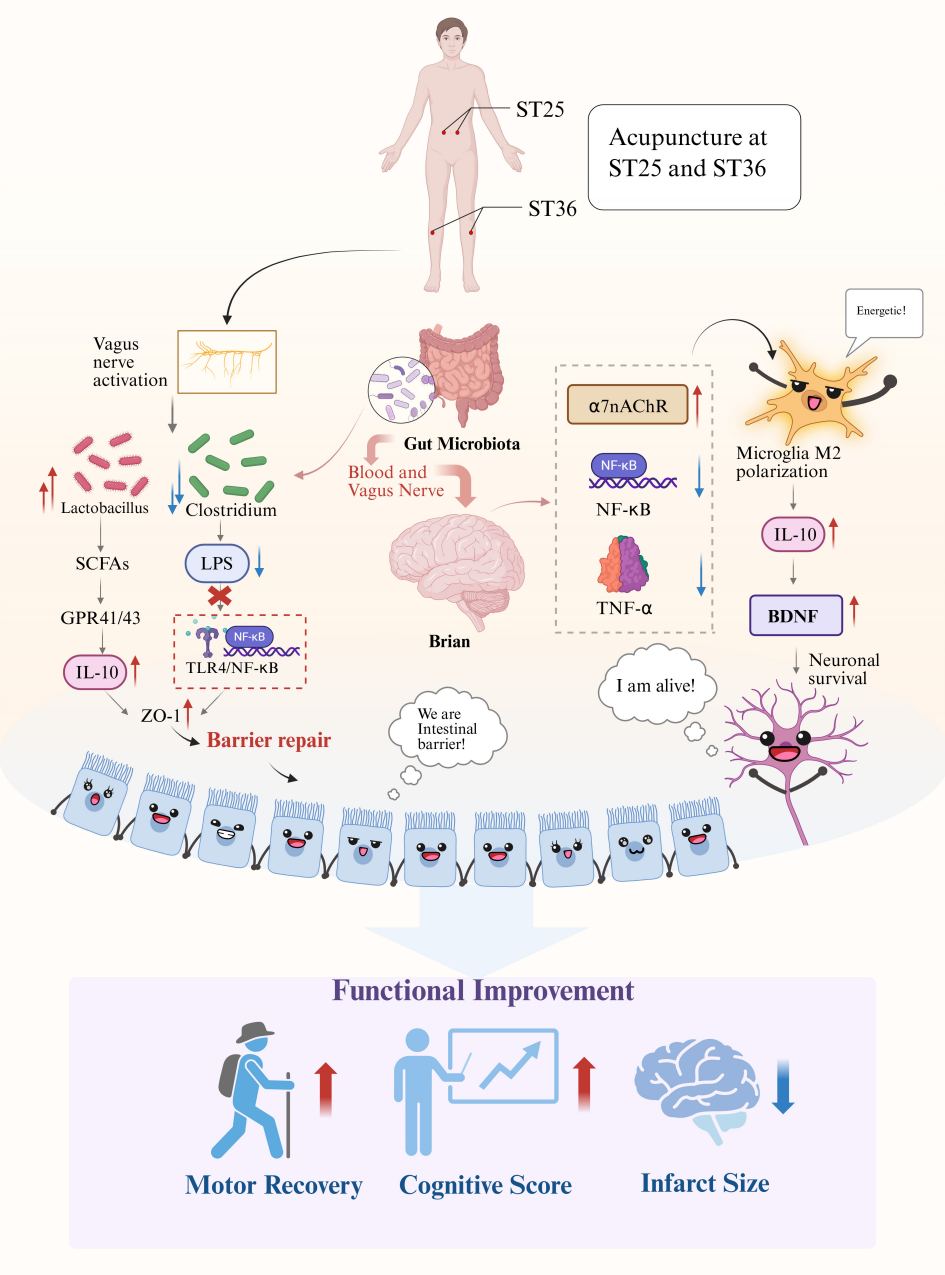
Acupuncture modulates gut-brain axis to promote stroke recovery.

Acupuncture shows significant potential for neurological rehabilitation, particularly in stroke recovery (35). Recent studies confirm that acupuncture not only restores microbial balance but also reduces inflammatory burden through gut-brain axis modulation in animal and clinical stroke models (36–38) (Table 1). Mechanistically, acupuncture acts through two key pathways: (1) the vagus nerve-mediated cholinergic anti-inflammatory pathway, which regulates cytokine balance via the α7 nicotinic acetylcholine receptor (α7nAChR) (39–41); and (2) the Toll-like receptor 4 (TLR4)/nuclear factor kappa B (NF-κB) pathway, which reduces neuroinflammation while enhancing neuroprotective metabolite production (39, 41, 42) (Figure 1). These metabolites enhance BDNF expression, promoting neuronal survival.

**Table 1:** Summary of studies on effects and mechanisms of acupuncture in modulating gut microbiota in stroke treatment.

**Study**	**Participants/animals**	**Disease**	**Treatment**	**Key findings**
Yan et al. (36)	Rats	Acute ischemic stroke	Electroacupuncture	Lactobacillus↑; Clostridium↓; reduced neuroinflammation via α7nAChR pathway
Cai et al. (37)	Rats	Poststroke depression	Acupuncture	Depression-like behavior↓; regulated gut microbiota and NLRP3 inflammasome in colon
Zhang et al. (38)	Mice	Ischemic stroke	Electroacupuncture + small extracellular vesicles	Modulated gut microbiota via brain-gut axis; reduced inflammation
Xian et al. (47)	Rats	Ischemic stroke	Acupuncture + herbal formula	Synergistic effects: Microbial diversity↑; improved metabolic profiles
Cai et al. (48)	Rats	Poststroke depression	Electroacupuncture	Altered fecal metabolites; beneficial bacteria (e.g., Bifidobacterium)↑
Li et al. (49)	Rats	Ischemic stroke	Electroacupuncture	Rapid tolerance via gut flora → melatonin receptor activation by indole-3-propionic acid
Li et al. (50)	Rats	Poststroke depression	Electroacupuncture	Altered gut microbiota; hippocampal neuroinflammation↓
Sun et al. (51)	80 patients	Poststroke constipation	Acupuncture vs. moxibustion	Lactobacillus↑, Bifidobacterium↑, Clostridium↓
Xiao et al. (53)	56 patients	Poststroke cognitive impairment	Acupuncture	Bifidobacterium↑; MoCA/MMSE scores↑; cognitive improvement linked to microbiota modulation

α7nAChR, α7 nicotinic acetylcholine receptor; NLRP3, NOD-like receptor family pyrin domain containing 3; MoCA, Montreal Cognitive Assessment; MMSE, Mini-Mental State Examination.

Yan et al. (36) demonstrated that acupuncture significantly modulated gut microbial composition and suppressed systemic inflammation in rats with acute ischemic stroke, suggesting its therapeutic potential via the microbiota–immune–brain axis. To further elucidate the molecular mechanisms, recent studies have shown that acupuncture activates the cholinergic anti-inflammatory pathway mediated by the α7nAChR, which inhibits pro-inflammatory cytokines such as interleukin-6 and tumor necrosis factor-alpha, while promoting the expression of the anti-inflammatory cytokine interleukin-10 in microglia through the Janus kinase 2/signal transducer and activator of transcription 3 (JAK2/STAT3) pathway (42, 43). Additionally, acupuncture regulates the TLR4/myeloid differentiation primary response 88 (MyD88)/NF-κB signaling cascade at multiple levels. In vascular dementia models, acupuncture significantly inhibits microRNA-93-mediated activation of the TLR4/MyD88/NF-κB pathway in microglia, thereby attenuating both neuroinflammation and oxidative stress (42). Similarly, studies in inflammatory bowel disease models have shown that electroacupuncture reduces intestinal inflammation by suppressing NLRP3 inflammasome activation and caspase-1-mediated pyroptosis through the same TLR4/NF-κB signaling pathway (44). These results underscore the multifaceted molecular actions of acupuncture in modulating inflammation across different disease contexts.

Acupuncture also strengthens gut barrier integrity by upregulating tight junction proteins (e.g., occludin, ZO-1) (45), and reducing endotoxin translocation (39). By inhibiting TLR4, acupuncture further protects the gut barrier. This dual modulation of microbiota and barrier function alleviates post-stroke neurological injury. Animal studies confirm that acupuncture restores microbial dysbiosis, increasing beneficial bacteria (e.g., *Lactobacillus, Bifidobacterium*) while reducing pathogenic bacteria (46). A combined metabolomics and microbiota sequencing study confirmed the synergistic benefits of acupuncture and herbal therapy, showing enhanced microbial shifts and metabolic regulation in ischemic stroke models (47). These results highlight acupuncture's role in immune regulation via the gut-brain axis.

Recent evidence has further elucidated the mechanisms by which electroacupuncture modulates gut–brain communication (Table 1). Cai et al. (48) reported that electroacupuncture significantly reshaped fecal metabolite composition and increased microbiota diversity in rat models of poststroke depression, suggesting that its antidepressant effects may be mediated via gut–brain axis regulation. Similarly, Li et al. (49) found that electroacupuncture enhanced the production of indole-3-propionic acid, a neuroprotective microbial metabolite that activates melatonin receptors, thereby promoting stroke resilience through the microbiota–neuroendocrine pathway. Additionally, Li et al. (50) demonstrated that electroacupuncture at Siguan acupoints reduced depression-like behaviors in poststroke rats by altering gut microbial composition and inhibiting hippocampal neuroinflammation. Collectively, these findings highlight the multifactorial pathways through which acupuncture exerts immunomodulatory and neuroprotective effects by targeting gut microbiota in the context of stroke rehabilitation.

In a randomized controlled trial (RCT) involving 80 post-stroke patients with constipation, acupuncture significantly alleviated bowel symptoms (51). These results are promising, but the small sample size and short follow-up period limit generalizability. The absence of a sham control group also complicates interpretation due to potential placebo effects. Participants were required to have a confirmed stroke diagnosis within the past 6 months, satisfy the Rome IV criteria for constipation, and have no recent exposure to antibiotics or probiotics (51). They were randomly assigned to either the acupuncture group (*n* = 40) or the moxibustion control group (*n* = 40), with well-matched baseline characteristics (51). The acupuncture group consisted of 29 males and 11 females (mean age: 58.27 ± 9.40 years), while the control group comprised 31 males and 9 females (mean age: 58.42 ± 9.85 years) (51). Most patients had ischemic stroke (35 vs. 37, respectively), and the average duration from stroke onset was 28.48 ± 7.53 days in the acupuncture group and 27.35 ± 8.48 days in the control group. Baseline National Institutes of Health Stroke Scale scores were 5.10 ± 4.57 and 5.77 ± 4.16, respectively (51). The treatment protocol involved acupuncture at Shuidao (ST28), and Guilai (ST29), administered every other day for 30 min per session over 3 weeks (51). Primary outcomes included changes in the constipation symptom scale (CSS) scores, frequency of spontaneous bowel movements, and defecation time (51). Secondary outcomes included patient-reported satisfaction and microbial community profiles, analyzed using 16S rRNA gene sequencing (51). Acupuncture significantly improved stool consistency (CSS score increased from 2.0 to 3.0), enhanced bowel movement frequency, and shortened defecation time (from 22.5 to 15 min). Microbiota analysis showed a decrease in *Clostridium* abundance from 47.72 to 40.67% after acupuncture, approximating the level observed in healthy controls (38.42%) (51). The relative abundance of *Lactobacillaceae* increased from 0.62 to 2.15%, and Bifidobacterium from 10.53 to 12.17%, reflecting a modest enhancement in probiotic populations (51). Although the increases in short-chain fatty acids (SCFAs)-producing bacteria such as *Ruminococcaceae* and *Eubacterium* were not statistically significant between groups, acupuncture was associated with a slight elevation in their abundance (51). Notably, the abundance of *Methanobacteriaceae*, which are linked to impaired gut motility, decreased substantially from 9.71‰ to 0.14‰ after acupuncture. These findings suggest that acupuncture may alleviate constipation, in part, by inhibiting methanogenic archaea.

Beyond stroke, acupuncture has been investigated for other gut microbiota-related conditions, such as leaky gut syndrome and inflammatory bowel diseases (52). Studies show that acupuncture enhances gut microbiota diversity, strengthens intestinal barrier function, and reduces permeability to harmful substances, thereby lowering gut inflammation (39, 52). In animal models of inflammatory bowel disease, acupuncture restores gut microbiota diversity, decreases intestinal permeability, alleviates inflammation, and boosts intestinal immune function (39, 52). Additionally, acupuncture enhances immune tolerance, reducing autoimmune responses and promoting gut health.

Acupuncture exerts neuroprotection through two primary neuroprotective mechanisms: First, it suppresses gut-derived inflammation through hypothalamic-pituitary-adrenal axis regulation (41), thereby protecting blood-brain barrier integrity (39, 40). Second, it enriches beneficial bacteria (e.g., *Lactobacillus, Bacteroides*), improving microbial diversity and promoting immune tolerance (39, 41, 45). Furthermore, acupuncture directly upregulates neurotrophic factors [e.g., brain-derived neurotrophic factor (BDNF)] and activates autophagy pathways, which collectively support neuronal regeneration (39, 41, 45). In a randomized controlled trial involving 52 patients with post-stroke cognitive impairment, *He-Mu* acupuncture combined with scalp acupuncture significantly improved cognitive function and regulated gut microbiota composition (53). After 3 weeks of treatment, Montreal Cognitive Assessment and Mini-Mental State Examination scores increased by 3.99 and 5.97 points, respectively, in the acupuncture group, significantly exceeding the improvements observed in the scalp acupuncture group (*P* < 0.01) (53). The abundance of *Bifidobacterium* and *Lactobacillus* increased significantly, by 2.17 and 2.67 log colony-forming unit (CFU)/g respectively, while *Escherichia coli* counts decreased from 7.55 to 4.62 log CFU/g (*P* < 0.05) (53). These results suggest that cognitive improvement may be partly mediated through gut microbiota modulation along the microbiota–gut–brain axis.

The interaction between acupuncture and the gut-brain axis represents a significant area of investigation in neuroscience research. Evidence suggests that acupuncture alters gut microbiota composition, particularly by increasing production of SCFAs like butyrate. These SCFAs activate G-protein-coupled receptors on immune cells, inhibiting NF-κB and reducing neuroinflammation while promoting anti-inflammatory M2 microglial polarization (29, 30, 45). This microbiota-driven immunomodulation affects both gut and brain immunity (39, 40). These mechanisms support acupuncture as a multifunctional therapy targeting gut-immune signaling and neural repair, providing a scientific basis for its use in stroke rehabilitation. However, a significant number of studies cited in this section are derived from overlapping research groups or are published in regional journals with limited international visibility. Although these studies offer valuable preliminary insights, their concentration within a limited set of academic sources raises concerns regarding potential publication bias. To improve the generalizability and scientific robustness of future conclusions, reviews should prioritize evidence from rigorously designed multicenter trials conducted in diverse populations and clinical contexts.

In summary, acupuncture modulates gut microbiota, immune responses, and neuroinflammation, making it a promising therapy for stroke recovery. Future RCTs should include blinded designs and longer follow-ups to assess the sustainability and clinical relevance of microbiota changes. Further research should explore its long-term effects on neurological recovery and gut-brain axis interactions.

## 6 Challenges and future directions

### 6.1 Methodological limitations

Although numerous studies suggest that acupuncture can modulate the gut microbiota and improve cognitive function, methodological inconsistencies limit the interpretability and reproducibility of findings. Common issues include small sample sizes, absence of randomization, inadequate blinding, and poorly defined control interventions, all of which reduce statistical power and compromise internal validity (54, 55). In addition, heterogeneity in study design—such as variability in acupoint selection, stimulation techniques, and treatment duration—complicates the comparison and synthesis of results across studies (54, 55). Furthermore, patient-level variability (e.g., disease severity, comorbidities, microbiota baseline composition) introduces confounding factors that obscure causal inferences (55, 56).

Moreover, several practical barriers hinder the clinical translation of acupuncture-based interventions. Prominent among these are the substantial variations in clinical trial design, such as differences in diagnostic criteria, acupoint selection, needling techniques, and treatment durations. Such variability undermines the reproducibility and comparability of study outcomes. Additionally, many trials lack statistical power due to small sample sizes, which increases the risk of false-negative findings and reduces the generalizability of results. The frequent absence of standardized outcome measures further complicates evidence synthesis. Another major limitation is the underrepresentation of diverse patient populations in clinical studies, especially across age groups, genders, ethnic backgrounds, and comorbidities, which limits the external validity of current evidence. A key limitation is lack of cost-effectiveness analyses comparing acupuncture with conventional therapies (e.g., probiotics or pharmacotherapy). These data are essential for guiding healthcare policies. Additionally, existing trials underrepresent diverse populations (e.g., elderly and ethnic minorities), highlighting the need for inclusive recruitment strategies to improve generalizability. Addressing these issues is essential to support the robust integration of acupuncture into evidence-based clinical frameworks for stroke rehabilitation. Moreover, dependence on a narrow body of literature—especially those originating from recurring research groups or lower-impact journals—may compromise the reproducibility and impartiality of current findings. Addressing this issue necessitates improved methodological transparency and the incorporation of evidence from internationally peer-reviewed, high-quality sources.

### 6.2 Mechanistic uncertainty

The biological mechanisms through which acupuncture exerts its therapeutic effects on the brain–gut axis remain insufficiently understood. While clinical and preclinical studies have reported significant changes in gut microbial diversity and metabolite production following acupuncture, it remains unclear whether these changes serve as direct mediators of neuroprotection or are instead secondary to systemic immune modulation (41). Clarifying these pathways is essential for establishing causality and identifying reliable biomarkers of response.

### 6.3 Barriers to clinical translation

Beyond experimental limitations, several practical challenges hinder the widespread clinical application of acupuncture-based interventions. First, acupuncture requires skilled practitioners, limiting scalability in resource-constrained settings (55). Second, existing studies rarely include long-term follow-up, and thus the sustainability of microbiota modulation and neurological benefits remains unknown beyond 6 months post-treatment (51, 53). Additionally, there is a lack of cost-effectiveness analyses and implementation frameworks to guide the integration of acupuncture into standard clinical practice. Scalability may be enhanced by exploring non-invasive, telehealth-compatible alternatives such as transcutaneous vagus nerve stimulation, which could extend access to underserved populations.

### 6.4 Research priorities and future directions

To overcome the above limitations, future research should pursue both mechanistic and translational objectives. At the mechanistic level, multi-omics approaches—including metagenomic sequencing, gene expression profiling, and single-cell Ribonucleic Acid sequencing—can elucidate the specific microbial taxa, host signaling pathways, and cell-type-specific responses implicated in acupuncture-induced modulation of the gut–brain axis (41, 57). Studies should also investigate neuro-immune interactions, particularly the vagus nerve–SCFA axis and the roles of microbial metabolites such as trimethylamine N-oxide and bile acids.

At the translational level, large-scale, multicenter RCTs with standardized protocols and diverse populations are urgently needed to validate efficacy, safety, and generalizability (54, 55). Parallel animal studies should explore molecular mechanisms in greater depth and facilitate biomarker discovery. Interdisciplinary collaboration among clinicians, neuroscientists, microbiologists, and health economists will be critical to advancing the integration of acupuncture into evidence-based strategies for stroke recovery and cognitive rehabilitation.

To address these challenges, future studies should prioritize large-scale, multicenter randomized controlled trials with standardized diagnostic criteria and uniform treatment protocols. Recruitment strategies should ensure demographic diversity to capture differences in treatment response across age, gender, and comorbidity spectrums. The use of adaptive trial designs and pragmatic approaches can improve the relevance of findings to real-world clinical practice. At the same time, developing consensus-based core outcome sets for acupuncture and gut microbiota modulation is crucial for promoting standardized reporting. These efforts will not only improve methodological rigor but also facilitate the accumulation of high-quality, generalizable evidence to support clinical decision-making and policy development.

## 7 Summary

Acupuncture has shown potential in stroke rehabilitation, particularly through its modulation of gut microbiota, which plays a key role in immune regulation and neurological recovery. Evidence suggests that acupuncture may enhance immune responses and promote neuronal function, aiding post-stroke recovery. While the exact mechanisms remain unclear, acupuncture's influence on the gut-brain axis is a promising area for further investigation. Future research should identify the molecular pathways involved, using techniques like metagenomics and neuroimaging to understand how acupuncture aids recovery. Translating these findings into clinical practice requires addressing two major limitations. First, optimal acupuncture parameters (e.g., frequency and duration) remain undefined. Second, it is unclear whether microbiota modulation directly mediates neuroprotection or is a secondary effect of reduced inflammation. Interdisciplinary collaboration is essential to resolve these issues.

As a non-pharmacological treatment, acupuncture offers a valuable alternative for stroke rehabilitation, especially for patients who experience side effects from conventional treatments. Its integration with modern therapies could provide a more holistic approach to recovery. Clinically, acupuncture should be considered a complementary therapy to enhance neuroprotection and immune modulation in stroke care, with further research needed to confirm its effectiveness. However, critical knowledge gaps persist, particularly concerning the long-term stability of microbiota changes and their potential causal role in neurological recovery. Future research should aim to identify robust microbial and immunological biomarkers predictive of acupuncture efficacy and to establish standardized protocols for acupoint selection, stimulation parameters, and treatment duration. Incorporating cost-effectiveness analyses and pragmatic implementation strategies into clinical trial designs will be essential for informing evidence-based clinical guidelines. These research priorities may ultimately facilitate precision rehabilitation approaches tailored to individual microbiota compositions and systemic inflammatory profiles.
